# Quality Evaluation of the Traditional Medicine Majun Mupakhi ELA via Chromatographic Fingerprinting Coupled with UHPLC-DAD-Quadrupole-Orbitrap-MS and the Antioxidant Activity In Vitro

**DOI:** 10.1155/2018/1035809

**Published:** 2018-03-06

**Authors:** Ayinuer Reheman, Haji Akber Aisa, Qing Ling Ma, Dilaram Nijat, Rahima Abdulla

**Affiliations:** ^1^University of Chinese Academy of Sciences, Beijing 100039, China; ^2^Key Laboratory of Plant Resources and Chemistry in Arid Regions, Xinjiang Technical Institute of Physics and Chemistry, Chinese Academy of Sciences, Urumqi, Xinjiang 830011, China; ^3^State Key Laboratory Basis of Xinjiang Indigenous Medicinal Plants Resource Utilization, Xinjiang Technical Institute of Physics and Chemistry, Chinese Academy of Sciences, Urumqi 830011, China; ^4^College of Traditional Uyghur Medicine, Xinjiang Medical University, Urumqi, Xinjiang 830011, China

## Abstract

By merging a high-performance liquid chromatography diode array detector (HPLC-DAD) method with high-performance thin-layer chromatography (HPTLC), an assay was developed for chemical fingerprinting and quantitative analysis of traditional medicine Majun Mupakhi ELA (MME), and constituent compounds were identified using HPLC coupled with UHPLC-DAD-Quadrupole-Orbitrap-MS method. In addition, the antioxidant capacity of MME was assessed based on the ability of components to scavenge radicals using in vitro method. Using a HPLC-DAD method with HPTLC easily validated the chemical fingerprinting results and quantified three characteristic components, namely, gallic acid (1), daidzein (2), and icariin (3), in commercial MMEs. The three compounds presented excellent regression values (*R*^2^ = 0.9999) in the ranges of the test and the method recovery was in the range from 100.49% to 100.68%. The fingerprints had 27 common characteristic peaks, of which 13 were verified by rapid UHPLC-DAD-Q-Orbitrap-MS analysis. In vitro antioxidant assays rapidly assessed and contrasted antioxidant activity or the free radical scavenging activity of the main polyphenolic classes in MMEs, and the antioxidant capacity was mostly affected by the presence of gallic acid. Thus, this study establishes a powerful and meaningful approach for MME quality control and for assessing in vitro antioxidant activity.

## 1. Introduction

The Uyghur medicine MME is administered in the form of a cream composed of* Epimedium brevicornum* Maxim,* Anacyclus pyrethrum* (L.) DC,* Lycium barbarum* L,* Cuscuta australis* R.Br,* Rhodiola crenulata* (Hook.f. et Thoms) H.Ohba,* Cinnamomum cassia* Presl,* Orchis morio* L.,* Polygonatum odoratum (Mill)* Druce, and* Crocus sativus* L. For a long time, the MME which has been used as an aphrodisiac and to treat both impotence and erectile dysfunction has been applied in clinical settings [1]. However, to date, there have been no empirical studies reporting its chemical composition, quality control standards, or the pharmacodynamic basis of its activities.

Many of the components of each herb in MME were reported to have excellent bioactivity, including aphrodisiac (PDE-5 inhibition), antiosteoporosis, phytoandrogenic, immunomodulatory, antioxidant, antifatigue, and antiviral activities and have been used to treat sexual dysfunction. A compound extract of* Epimedium*,* Rhodiola*,* Cuscuta australis* R.Br, and* Lycium barbarum* L. contains flavonoids, glycosidic constituents, phenylpropanoids, phenylethanoids, flavonolignans, glycosides, proanthocyanidins, and polysaccharides [2–6].

However, the components and bioactivity of MME as a whole are unclear. It is necessary to clarify the formula components because the effects are not due to the addition of each individual herb in MME, but involve the synergistic effects between several components of each herb. Therefore, it is necessary to clarify the MME components and evaluate the quality standard.

In this study, an analysis of the chromatographic fingerprint of MME using HPLC combined with HPTLC and mass spectrometry was conducted in parallel. The chemical fingerprint builds a characteristic chemical profile of an MME or a material that contributes to its identification.

In recent years, great advantages in specificity and sensitivity have been demonstrated by the application of liquid chromatography combined with mass spectrometry (LC/MS), and effective chromatographic separation for qualitative analysis of compounds within herbal extracts can be applied to phytochemical studies [7–10].

Moreover, some MS techniques, such as TOF-MS (Q-TOF), Q-Orbitrap-MS, and IT-MS, have enabled people to obtain abundant structural information about interesting analytes. At present, study of herbal medicine quality often used a quantitative analysis of multiple components and an HPLC fingerprint [11–13]. Subsequent analysis employing instrumental techniques (such as mass spectrometry) [14] or fingerprinting (as analysis of the thin-layer chromatogram) can be used to achieve compound identification [15].

TLC (thin-layer chromatography) is a technique that is commonly used to screen low molecular weight compounds in complicated pharmaceutical, environmental, and food samples [16] and has taken precedence over other chromatographic approaches such as GC (gas chromatography) and HPLC because of its flexibility, cheapness, accessibility, and simplicity. As sophisticated instrumentation and high-performance adsorbent layers have been developed for sample analysis and chromatogram and derivatization evaluation, HPTLC and chromatogram development have become fairly popular. As an effective and rapid method for analysing complicated mixtures, among the many HPTLC applications, its utilization is of particular interest in fingerprint analysis [17].

Recently, studies have indicated that oxidative stress leads to the formation of radicals that can cause several diseases, and thus, the antioxidant activity of a medicine is an important factor in the treatment of disease [18, 19]. There are no reports of systematic testing for antioxidant activity of MME. Although antioxidant activity is commonly determined using spectrophotometric assays, the disadvantage of these spectrophotometric methods is that they measure the antioxidant capacity of the entire extract instead of the antioxidant capacity of the individual components present in the extract [20, 21]. When using TLC, many samples can be run at the same time on the same plate and thus under the same experimental conditions, making analysis times short and reducing the cost. Previously, antioxidant screens of plant extracts [22], herbal extracts [23], marine bacteria [24], and wine extracts [25, 26] have utilized TLC coupled with a nitrogen radical 1,1-diphenyl-2-picrylhydrazyl (DPPH^•^) assay in situ. A mature plate is dipped or sprayed in DPPH^•^ free radical solution in alcohol to produce white TLC-DPPH^•^ spots on a pink background, which represent the presence of active antioxidant compound [23].

2,2′-Azino-bis(3-ethylbenzothiazoline-6-sulfonic acid) (ABTS^*∗*+^) coupled with the DPPH^•^ free radical scavenging method is usually utilized to assess the antioxidant activity of medicinal herbs [27–30]. A comprehensive evaluation of radical scavenging activity and antioxidant activity could utilize these two approaches.

To date, there have been no reports in the literature concentrating on comparative analysis of the chemical fingerprint and antioxidant activity of the MME using a chromatography technique. Thus, the purpose of this study is to use both HPTLC fingerprinting and HPLC fingerprinting coupled with mass spectrometry to identify the chemical profile and evaluate the quality standard of the MME compound. In addition, the fast and simple TLC method coupled with postchromatographic derivatization with either FeCl_3_ or DPPH^•^ free radical and ABTS^*∗*+^ was used to screen the antioxidant activity of the MME.

## 2. Materials and Methods

### 2.1. Chemicals and Material

HPLC grade methanol from Merck (Darmstadt, Germany), MS grade formic acid from Sigma-Aldrich (Steinheim, Germany), and HPLC grade acetonitrile from Merck (Darmstadt, Germany) were purchased. A Milli-Q system provided ultrapure water (Millipore, Bedford, MA, USA). Reference compounds for icariin (batch number: 110737-201516, purity: >98%), gallic acid (batch number: 110831-201605), and daidzein (batch number: 11502-20-0402) were obtained from the Chinese Food and Drug Accreditation Institute. 1,1-Diphenyl-2-picrylhydrazyl (DPPH^•^) free radical was obtained from Munich, Germany, and 2,2′-azino-bis(3-ethylbenzothiazoline-6-sulfonic acid) ABTS^*∗*+^ free radical was from Sigma. All other chemicals and solvents used were of analytical grade. Normal phase silica gel 60 F_254_ HPTLC glass plates with a size of 20 × 10 cm were used to perform separation (Merck, Darmstadt, Germany).

### 2.2. Plant Samples and Crude Drug

*Epimedium brevicornum* Maxim (batch number: 201609003),* Anacyclus pyrethrum* (L.) DC (batch number: 201608003),* Lycium barbarum* L. (batch number: 201607003),* Cuscuta australis* R.Br (batch number: 201607003), and other plants were purchased from the Hospital of Traditional Uyghur Medicine in Xinjiang. Anwar Talip, the director of the pharmaceutical department, identified them as authentic medicine.

*Preparation of the MME*. In the prescription, apart from the* Crocus sativus* L., other herbs were washed, dried, and crushed, over 100 mesh sieve, mixed (MMEs crude drug), and added to refined honey (honey temperature reduced to room temperature can be added), then stirred after adding* Crocus sativus* L. fragmentation, and continuously mixed and the cream was gotten.

Ten batches of MMEs crude drug (batch numbers 20161213, 20170112, 20170122, 20170210, 20170220, 20170228, 20170315, 20170325, 20170328, and 20170415) were provided by Xinjiang Institute of Biomedical Innovation.

### 2.3. HPLC-DAD Analysis

#### 2.3.1. Sample Preparation

The MMEs crude drug was precisely weighed and extracted using 25 mL of 50% ethanol in an ultrasonic water bath for 30 minutes at room temperature and then centrifuged at 8000 r/min for 8 minutes to obtain the supernatant. Before analysis, a 0.22 *μ*m filter membrane was used to filter the sample solutions.  Table 1 is the similarity of ten batch samples.

#### 2.3.2. HPLC-DAD: Equipment and Quantification Conditions

An Agilent 1260 series HPLC instrument (Santa Clara, CA, USA) equipped with an Agilent DAD detector (All Tech, Lexington, KY, USA) was used to obtain HPLC fingerprints. A Thermo C_18_ column (250 mm × 4.6 mm, i.d., Agilent) was used for analyses. For separation, a column temperature of 30°C, a flow rate of 1.0 mL/min, and an injection volume of 10 *μ*L were used.

The optimized elution conditions were as follows: (C) acetonitrile and (D) 0.3% formic acid in water were used as the mobile phase. A linear gradient of 0%–7% (C) (0–15 min), 7%–20% (C) (15–20 min), and 20%–43% (C) (20–40 min) with wavelength conditions of 273 nm (0–20 min), 360 nm (21–30 min), and 270 nm (31–40 min) was applied.

#### 2.3.3. HPLC-DAD Method Validation

After establishing the optimal conditions, the quantitative analysis method was validated in terms of the China Pharmacopeia guidelines and recommendations. The validation of the method covers the linearity, repeatability, stability, recovery, and precision.

*(1) Linearity*. A series of normalized solutions containing 3 marker compounds was freshly prepared using six chemical markers at various concentrations and examined under the optimal separation conditions with UV detection. Analysis of every concentration was performed in triplicate. Linear regression was established by drawing the complete peak area (*Y*) of the chromatogram versus the corresponding concentration of the injected normalized solutions (*X*).  Table 2 presents the summary of the calculated results. The regression equations were computed in the format of *y* = *ax* + *b*, in which *x* represents the compound concentration (*x*, *μ*g/mL) and *y* represents the peak area.

*(2) Precision, Repeatability, Stability, and Recovery of MME*. The precision (reproducibility) of this method was determined by computing the RSD (relative standard deviation) of repeated injections of the normalized solution. Six repeated injections determined the precision, for both peak areas (PAs) and retention times (RTs). The RSD for every compound PA was computed.

To assess repeatability, 8 aliquots from the same batch of the test sample were weighed accurately, and the same preparation steps and method described in Section 2.3.2 were performed, according to the requirements of the content detection (Section 2.3.2), to determine the gallic acid, daidzein, and icariin peak area and to calculate the gallic acid, daidzein, and icariin contents as well as the RSD value.

Stability was examined by evaluating 7 aliquots taken from the same batch of test sample. Solutions at 3 different concentrations were evaluated at time intervals of 0, 2, 4, 6, 8, 12, and 24 h at room temperature. The stability was determined based on the relative standard deviation.

To investigate recovery, accurately weighed samples (0.050 g) with a known content (gallic acid, daidzein, and icariin labelled 0.121%, 0.0407%, and 0.63%, resp.) were prepared. A total of 6 duplicate samples were placed in a 10 ml volumetric flask, according to the content of gallic acid, daidzein, and icariin in the sample, added in the amount of 1 : 1 gallic acid, daidzein, and icariin reference substance solution. Then, 50% ethanol was added, and the samples were ultrasonicated (250 W, frequency 40 kHz) for 30 minutes and allowed to cool to a constant volume according to a scale, and the above method was used to determine gallic acid, daidzein, and icariin content and calculate the recovery.

### 2.4. Quantitative Determination of the Three Marker Compounds in MME

In this study, to simultaneously determine the three main components extracted from varied batches of MME, the proposed HPLC-DAD method was applied by contrasting the online UV spectra and retention times with those of the standards. The determination of every sample was done in triplicate.

### 2.5. HPLC Conditions for Fingerprinting and Similarity Analysis

The mobile phase consisted of (C) acetonitrile and (D) 0.3% formic acid in water. The optimized elution conditions were as follows: linear gradient 10% C (0–10 min), 10%–43% C (10–55 min), 43%–55% C (55–70 min), 55%–80% C (70–75 min), 80% C (75–85 min), and then back to 10% C in 1 min and isocratic 10% C for 15 min, with the flow rate set at 1.0 mL/min and an injection volume of 10 *μ*L. The wavelength conditions were as follows: 0–20 min (273 nm), 20–25 min (290 nm), 25–30 min (360 nm), and 30–90 min (270 nm). All determinations were made in triplicate with data analysed using Similarity Evaluation System for Chromatographic Fingerprint of Traditional Chinese Medicine software (Version 2004 A) that the Chinese Pharmacopeia Committee developed.

### 2.6. UHPLC-DAD-Quadrupole-Orbitrap-MS Analysis

An Agilent 1200 series HPLC system was used to identify the compounds in MME and included a DAD (diode array detector) and a G1311A quaternary solvent delivery system.

An Orbitrap Q-Exactive high-performance benchtop MS analyser system obtained from Thermo Fisher Scientific (Bremen, Germany) combined with an ultrahigh-pressure liquid chromatography (UHPLC) system (Thermo Scientific Accela™, Thermo Fisher Scientific, Bremen, Germany) with a Thermo RP C_18_ column (250 mm × 4.6 mm, i.d., Agilent) was used for analysis.

Chromatographic separation conditions were as follows: 35°C, column temperature; a mobile phase system consisting of solvent A (0.3% formic acid/water H_2_O) and solvent D (acetonitrile), with a linear gradient of 0–10 min (2%–10% D), 10–55 min (10%–43% D), 55–70 min (43%–55% D), 70–75 min (55%–80% D), 75–85 min (80% D), 85-86 min (80%–100% D), and 86–96 min (100% D).

The DAD wavelength was set at an acquisition range of 200–600 nm, with an injection volume of 10 *μ*L and a flow rate of 1.0 mL/min. The scan acquisition range was *m*/*z* 100–1500 at 70,000 (FWHM), with a resolving power of *m*/*z* 200. The positive mode was used to obtain a spectral speed of 3 Hz with an HESI (heated electrospray) ion source. The following were the mass spectrometric parameters: 300°C as the heater temperature; 3.8 kV as the electrospray voltage; 350°C as the capillary temperature; nitrogen (N_2_) as the auxiliary and sheath gas; helium (He) as the collision gas. The flow rate of the auxiliary gas and the sheath gas pressure were 10 arb and 30 psi (1 arb = 0.3 L/min), respectively. The collision energy (CE) was set between 30 and 70 eV, which generated more information about compound structure. Taurocholic acid (2 ng/*μ*L) injected using a syringe pump with 10 *μ*L/min as the flow rate was used to calibrate the mass analyser. Analyst QS 2.0 software was used to perform data collection and processing.

### 2.7. High-Performance Thin-Layer Chromatography

#### 2.7.1. HPTLC Fingerprint

The 50% ethanol dried crude drug (10 batches), obtained as explained above, was boiled in water for 30 minutes, hot-filtered, extracted with ethyl acetate, and dissolved in methanol. The filtered solutions were applied to glass plates 20 cm × 10 cm (Merck, Darmstadt, Germany) with a glass-backed layer of silica gel 60 F_254_ (2 *μ*m thickness).

We washed the plates with methanol before use and dried them for 2 hours at 105°C. A CAMAG (Muttenz, Switzerland) 10-sample application with 25 *μ*L syringes linked to a nitrogen tank realized was used for this purpose. The following were the operation conditions: 10 s/*μ*L as the syringe delivery speed; 15 *μ*L as the injection volume; 6 mm as the bandwidth; 15 mm as the start position; and 8 mm as the distance from the plate bottom. An ethyl acetate-formic acid-glacial acetic acid-water (16 : 1 : 1 : 1, v/v/v/v) mixture was used to saturate the HPTLC plates in the automatic developing chamber ADC2, which reproducibly produced the same 30-minute mobile phase at room temperature. Finally, a TLC plate heater (CAMAG) was used to warm the plates for 5 minutes at 105°C until there was a distinct colour of MME on the plate. A UV viewer cabinet (CAMAG) was used to examine the fluorescence image under 365 nm UV light, which was obtained with a Digistore 2 documentation system (CAMAG). The reflection mode employed a 366 nm excitation wavelength, and the exposure time was 3 s.

#### 2.7.2. In Vitro Antioxidant Activity and Chromatographic Band Visualization

In brief, 10–80 *μ*L of MME was applied to 20 cm × 10 cm silica gel HPTLC plates (Art. 105641, Merck, Darmstadt, Germany) as an 8 mm band by utilizing an automatic TLC sampler 4 (ATS4, CAMAG, Muttenz, Switzerland). An ethyl acetate-formic acid-glacial acetic acid-water (16 : 1 : 1 : 1, v/v/v/v) mixture was used to develop plates in a saturated vertical twin chamber for 30 min, until the bands reached a distance of 70 mm. A hairdryer was used to dry the developed plates for 5 minutes, and a TLC Plate Heater III (CAMAG) was used to heat the plates for three minutes at 105°C, which were immediately dipped into 0.5% solutions of ABTS^*∗*+^ and DPPH^•^ in an hydrous ethanol using a Chromatogram Immersion Device III (CAMAG). UV light was applied to the sample at 366 nm, with white light below and above the plate. We photographed the developed plates before and after they were derivatized with either 0.4% w/v DPPH^•^ solution or ABTS^*∗*+^ solution. Before photographing, plates with derivatization of ABTS^*∗*+^ and DPPH^•^ solution were placed in a dark environment for 30 minutes. The reproducibility between the plates and high quality images was ensured by fixing the parameters captured using the winCATS imaging software. Video Scan Digital Image Evaluation software (2003, CAMAG, Muttenz, Switzerland) was used to perform quantitative analysis of HPTLC and was set to identify fluorescent bands. To process images further, the photos were stored in TIF file format.

## 3. Results and Discussion

### 3.1. Optimization of HPLC Chromatographic Conditions

We investigated the HPLC conditions (detection wavelength and mobile phase) for optimal chromatographic separation. In addition, we investigated how the composition of the mobile phase influenced chromatographic separation. Acetonitrile was used as the organic mobile phase due to its low viscosity, which lowers the system pressure, and its high elution of various polar compounds within herbal medicine [31, 32].

Finally, the proposed mobile phase of acetonitrile 0.3% formic acid (v/v) produced the most effective HPLC results. A DAD full wavelength scan (190–400 nm) was used to select target compound wavelengths in MME. Most chemical constituents of MME had strong UV absorbance. Therefore, 273 nm was the maximum absorption wavelength (for gallic acid), being 270 nm for icariin and 260 nm for daidzein; thus, these wavelengths were selected as the detection wavelengths; the following were the optimal HPLC conditions applied in this study. The mobile phases were composed of mobile phase C (acetonitrile–0.3% formic acid, 7 : 93, v/v–43 : 57 v/v) with a gradient program. To obtain a better peak number and peak resolution, we monitored the UV absorbance at different wavelengths of the same channel with a DAD. The injection volumes for all standard and sample solutions were 10 *μ*L.

### 3.2. Method Validation of Quantitative Analysis

#### 3.2.1. Linearity of Calibration Curves

Normalized stock solutions of gallic acid, daidzein, and icariin were prepared and dissolved in methanol. A series of concentrations of normalized solutions was used to plot the calibration curves, based on the PA or the ratio of the absorbance of the examined reference and that of the internal standard versus their concentrations.  Table 2 shows the correlation coefficient and regression equation. Reasonable linear regression values (*R*^2^ ≥ 0.9999) were obtained in the ranges of 5.568~33.408 (*μ*g/mL), 2.368~14.208 (*μ*g/mL), and 20.640~123.840 (*μ*g/mL) for all analyses.  Table 2 clearly demonstrates that the analytical method used was acceptable and had good sensitivity.

#### 3.2.2. Precision, Repeatability, Stability, and Recovery

Six replicates of 3 normalized solutions were used to assess the precision and RSD (relative standard deviation) of RRTs and RPAs, which were less than 0.005%, demonstrating the efficiency of the instrument. To examine the stability, recovery, and repeatability, 6 replicates of the same sample solutions were prepared and analysed. All the precision data (relative standard deviation) were below 1%, which revealed the acceptable accuracy and precision of this approach.

As shown in Tables 3(a) and 3(b), the relative standard deviations, which were used for evaluating the stability of the method, were below 1%, indicating that sample solutions that had been prepared and maintained for 24 hours at room temperature were relatively stable. In the recovery examination, a known amount of 3 standards was put in 50.3 mg of powder from ten batches of the same samples, and then, the standards were extracted and analysed using the same steps. Tables 3(a) and 3(b) show the RSDs.

### 3.3. Quantitative Determination of the Three Marker Compounds in MME

HPLC-DAD method put forward was successfully applied to simultaneously determine the 3 markers within MMEs in this research. The DAD profiles and retention time confirmed the marker compound peak identity in chromatograms.  Table 4 presents a summary of marker compound content in the ten samples. We did not observe any significant variances between the content of the same markers from varied samples. For instance, the icariin content ranged between 0.59% and 0.63%.

Nevertheless, there was no significant difference between each marker's contents determined between the sample and relative standard deviation values of 0.0284%, 0.1440%, and 0.0811%, revealing the small variations among their quality. This result can possibly be explained by the consistency both in the manufacturing processes and in the herbal materials applied. It is noteworthy that chromatographic fingerprints may quite fully monitor the quality consistency. No matter what, therefore, more importance should be attached to MME's quality consistency to guarantee its clinical effect. Our results accorded with those of previous studies. Compared to the chromatographic fingerprint alone, the chromatographic fingerprint coupled with quantitative analysis to determine marker compounds acts as an intermediate tool for evaluating the quality consistency of herbal preparations [33–35].

**Figure 1:**
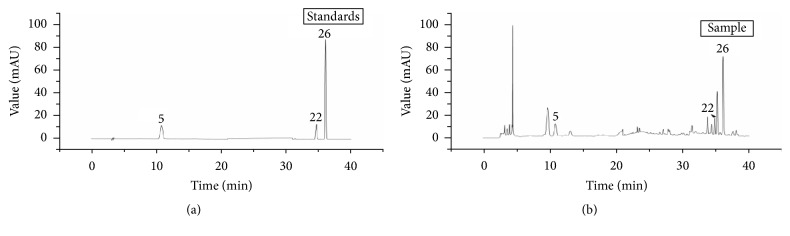
HPLC chromatograms of samples and standards.

**Figure 2:**
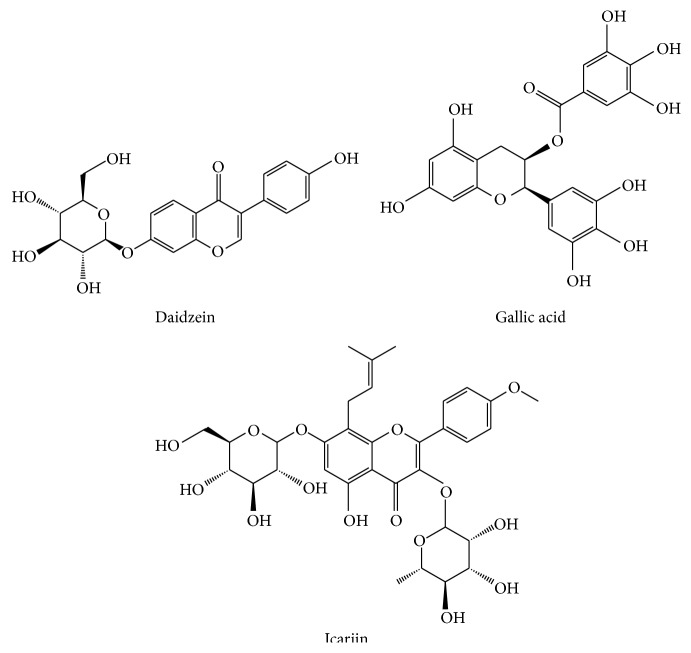
The chemical structure of the three marker compounds.

Quantitative analysis of the total icariin, gallic acid, and daidzein content (Figures 1 and 2) applied the verified conditions. The constructed quantitative analytical approach was used to analyse 10 batches of the samples, and Table 4 presents calculations of the contents. The results indicated that each compound had quite different content in ten batches of MME.

### 3.4. HPLC-DAD Fingerprinting

#### 3.4.1. Evaluation of the HPLC Fingerprints

**Figure 3:**
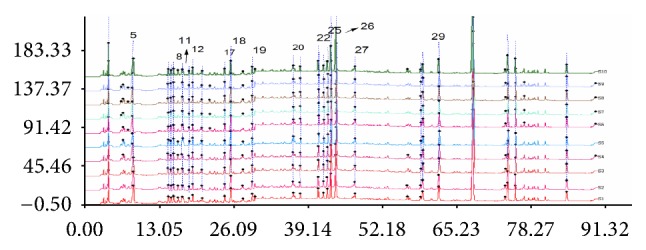
HPLC fingerprint of 10 sample batches (1–10 represent ten sample batches of MME).

Chromatographic fingerprints were generated for 10 MME samples, and 27 peaks were discovered in each separate sample (Figure 3). The simulative median chromatogram of MME had 13 well resolved “characteristic peaks.” Icariin (peak 26) was a vital bioactive component of MME, with a consistently high content and a suitable retention time. Although there was a slight difference between the absorption intensities of some peaks in some samples, the chromatographic profiles were generally consistent. We computed each chromatograph's similarity against the simulated median chromatogram: the similarities of S1–S10 were 0.998, 0.998, 0.999, 0.998, 0.981, 0.997, 0.998, 0.998, 0.998, and 0.997. The similarity values were all in the range of 0.981 and 0.999, indicating the presence of similar chemical components in the samples, despite the varied batch amounts, and revealing the feasibility and usefulness of the proposed method for fingerprinting.

### 3.5. UHPLC-DAD-Quadrupole-Orbitrap-MS Analysis

#### 3.5.1. Peak Detection

**Figure 4:**
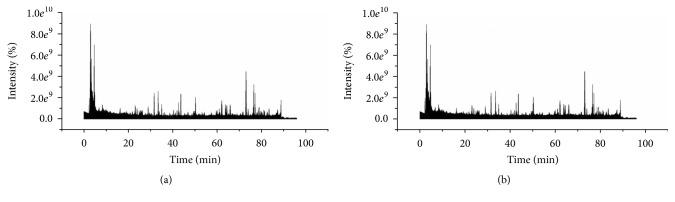
Sample analysis using UHPLC-DAD-Quadrupole-Orbitrap-MS ((a) negative ion method; (b) positive ion method).

To qualitatively express the chemical ingredients in MME fingerprints, their structures were identified using the online Quadrupole-Orbitrap-MS technique. For MS conditions, we tried both negative and positive ion modes. Our data showed that, compared with positive mode, negative mode was more sensitive in detecting the compounds in MME. Moreover, the cone and capillary voltage were set at 40 V and 3000 V, respectively, to balance the component ionization in these samples. As shown in Figure 4, good detection was achieved for 27 compounds, and numerous minor compounds were observed.

#### 3.5.2. Identification of the Compounds

In total, we identified 13 compounds from 27 peaks in MME; first, the components were confirmed by comparing the base peak chromatograms of MME and its individual herbs. Then, we compared the precise mass (mass error of less than 5 ppm) and retention time with previous reports and standards to preliminarily identify the components. Finally, component chemical structures were confirmed using fragment ions. Figure 4 shows the base peak chromatograms of MME in negative and positive ion modes, and the constituent information is shown in Table 5; Table 5 lists the 13 most common compounds identified.

Compound 5 (*t*_R_ = 9.23 min) gave an [M − H]^−^ ion at *m*/*z* 169.01308 [M − H]^−^ (C_7_H_6_O_5_) in the full-scan mass spectrum and abundant fragment ions at *m*/*z* 125.02302 [M − H-44]^−^ in the MS^2^ experiment, resulting from the loss of carbon dioxide (CO_2_). Based on the similarity in fragmentation patterns and retention time with the standard reference, the fragmentation information led to the conclusion that compound 5 was gallic acid [36].

Compound 22 (*t*_R_ = 40.28 min) had an [M + H]^+^ ion at *m*/*z* 255.06542 (C_15_H_10_O_4_) in the positive full-scan mass spectrum. The fragment ions were 195.04512 [M − CH_2_O-CO]^−^. According to the fragmentation patterns published in a previous report [37] and standard reference, we concluded that compound 22 was daidzein.

Compound 26 (*t*_R_ = 43.90 min) displayed an [M + H]^+^ ion at *m*/*z* 677.24431 (C_33_H_40_O_15_) in the positive complete scan mode. Characteristic fragment ions were observed at *m*/*z* 531.18730, 369.13281, and 313.07016. The ion of *m*/*z* 369.1336 was a typical fragment for anhydroicaritin aglycone. The ion at *m*/*z* 313.07016 was formed by further loss of C_4_H_7_ from the ion of *m*/*z* 369. For the prenylated flavonoids, the glucose substituted at the 3-O position is lost with more ease compared with that substituted at the 7-O position according to the characteristic fragmentation pathways. A base peak at *m*/*z* 531.18730 was given by the MS^2^ spectrum, which corresponded to hydroicaritin aglycone bearing an O-linked monosaccharide substituent. Based on the authenticated standard and a previous report [38], it was concluded that compound 26 was icariin.

Compounds 8, 11, 12, 17, 18, 19, 20, 25, 27, and 29 were unambiguously identified as salidroside, catechin, chlorogenic acid, rutin, ferulic acid, hyperoside, luteoloside, quercetin, apigenin, and kaempferol, respectively, by contrasting the fragment patterns, retention times, UV, and MS data with those of authentic standards.

### 3.6. High-Performance Thin-Layer Chromatography

#### 3.6.1. HPTLC Fingerprint

As complex mixtures, extracts of natural products comprise a wide range of compounds. The assay of quality and authenticity usually takes their thin-layer chromatographic profile into consideration [39].

Nevertheless, the HPTLC methods are less costly and provide colourful and vivid images for parallel contrast. Moreover, video recording is suitable for efficient and fast TLC chromatogram collection because of the uniform surface lighting, short scanning time, multichannel scanning competency, and strong optical resolution. MMEs contain more medicinal herbs and their chemical composition is also more complex. A huge number of samples, such as ten batches of samples, must be used to accurately determine the characteristic identity of compounds to disclose distinctions among complicated mixtures. HPTLC fingerprint analysis was used to search a characteristic set of numerous MME samples.

**Figure 5:**
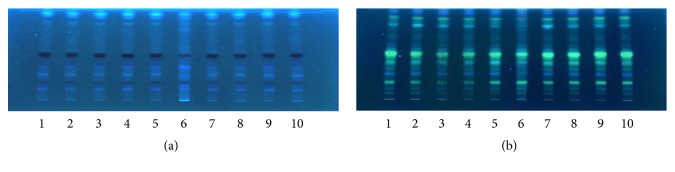
Developed TLC plates of the MME; (a) under UV 366 nm; (b) after derivatization (10% sulfuric acid in ethanol); from left to right (1–10 represent the ten sample batches (spot volume 15 *μ*L)).

**Figure 6:**
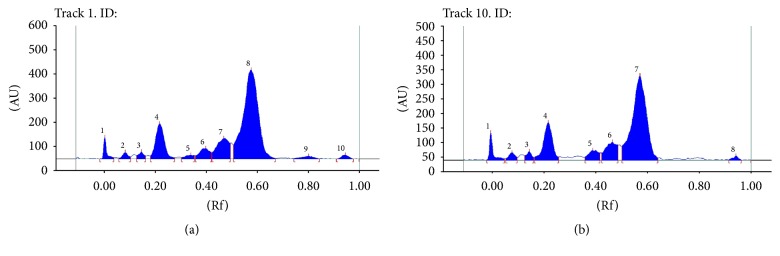
HPTLC fingerprint profile of MME; (a) and (b) represent samples 1 and 4, respectively.

**Figure 7:**
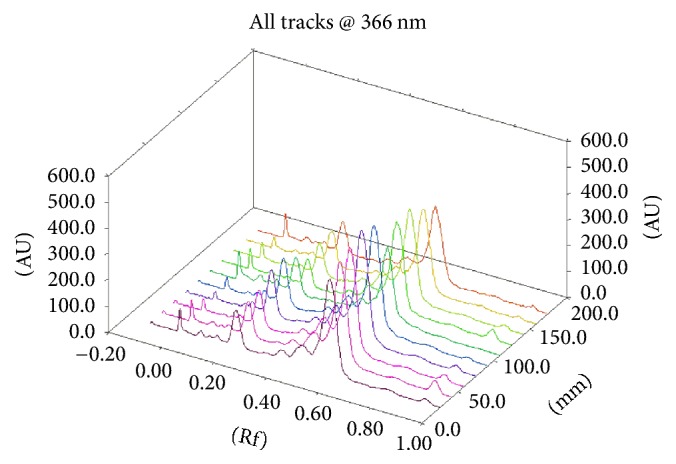
3D chromatogram profile (A); from bottom to top, 1–10 represent the ten sample batches (spot volume 15 *μ*L).

**Figure 8:**
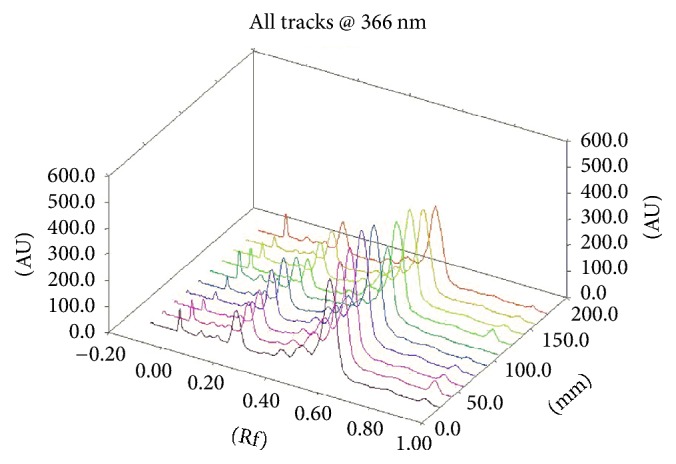
3D chromatogram profile after derivatization (B); from bottom to top, 1–10 represent the ten sample batches (spot volume 15 *μ*L).

Assessment of the chromatographic pattern of the MME samples analysed was on the basis of utilization of optimized HPTLC conditions with regard to chromatographic behaviour. Even though TLC analysis employed a great variety of adsorbents, chromatographic techniques, and solvent systems, the association of a silica gel stationary phase with developing solvents, including mixtures of 3, 4, or even 5 solvents of diverse polarities, is the most common. Typically, there is high use of a normal phase system for separation. Furthermore, glacial acetic acid and formic acid are put in the mobile phase for suppressing ionization of the acidic groups and promoting chromatographic band shape. A distinction in the chemical constitution of MMEs was revealed by a visual test of the acquired HPTLC chromatograms (shown in Figures 5, 6, 7, and 8). Phenolic compounds were abundant in samples of MME, whose pattern is dominated by orange and blue bands. It has been reported that individual herbs in the extract also contain phenolic compounds.

Quality evaluation is provided with excellent input data by HPTLC fingerprint chromatograms, such as colourful pictures, including those acquired for samples of MME.

#### 3.6.2. In Vitro Antioxidant Activity and Chromatographic Band Visualization

It has been discovered that TLC combined with biodetection is particularly helpful in identifying and detecting natural antioxidants. This method first separates the components of natural mixtures on a TLC plate as the adsorbent bed, and subsequently, ABTS^*∗*+^ or DPPH solutions are applied by spraying or dipping the plates into the solution [40].

In our work, HPTLC combined with a postderivatization DPPH^•^ assay and ABTS^*∗*+^ assay was successfully employed to screen MMEs for polyphenolic content (gallic acid) and antioxidant activity. A direct ABTS^*∗*+^ and DPPH^•^ assay was used to assess the free radical scavenging activity of the MME.

As a stable free radical with a deep pink colour, DPPH^•^ becomes white if the antioxidants present in the sample reduce it. Thus, antioxidants in the sample emerge as white spots and contrast with the pink background above the plate. ABTS^*∗*+^ is a catalase substrate, and ABTS/ABTS^*∗*+^ has a redox potential of 0.68 V, which is prone to electron transfer shift, and generates the stable green free radical ABTS^*∗*+^.

However, our study indicated that there are much more potent antioxidants in the investigated samples. Higher amounts of samples contained other antioxidants that showed higher free radical scavenging activity, as evidenced by the white band intensity after derivatization with DPPH^•^. The method developed in this work provides an edge over existing methods used to screen MME for antioxidant activity because it is possible to quantify antioxidant activity for individual compounds in the extract mixtures. This work also clarifies the versatility and flexibility of a normalized HPTLC system as a useful tool in the drug discovery process. The method developed in this work can also be used for the bioassay guided isolation of unknown natural antioxidants in extract mixtures and the subsequent identification of components utilizing postchromatographic mass spectrometry analysis techniques.

**Figure 9:**
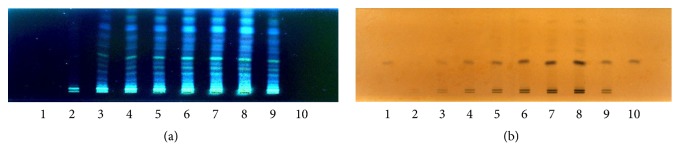
Developed TLC plates of the MME and gallic acid after derivatization. (a) After derivatization of 10% sulfuric acid in ethanol; (b) after derivatization of 1% ferric chloride; from left to right (1, 10 gallic acid (spot volumes 2 *μ*L and 5 *μ*L); 2–9 different concentrations of the extract (spot volumes 2 *μ*L, 8 *μ*L, 15 *μ*L, 25 *μ*L, 40 *μ*L, 60 *μ*L, 80 *μ*L, and 25 *μ*L)).

**Figure 10:**
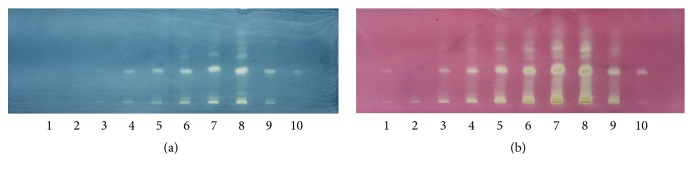
Developed TLC plates of the MME and gallic acid after derivatization. (a) After dipping in ABTS^+^ solution; (b) after dipping in DPPH solution; from left to right (1, 10 gallic acid (spot volumes 2 *μ*L and 5 *μ*L); 2–9 different concentrations of the extract (spot volumes 2 *μ*L, 8 *μ*L, 15 *μ*L, 25 *μ*L, 40 *μ*L, 60 *μ*L, 80 *μ*L, and 25 *μ*L)).

The comparison between the colour intensity and area of white bands of crude drug acquired by gallic acid normalized solutions after spraying with an ethanolic DPPH^•^ solution (Figure 10) was used to assess the free radical scavenging activity degree within extracts in gallic acid. Some authors claim that there is no correlation between the phenolic content and the radical scavenging capacity, but in this work (Figure 9), we confirmed that free radical scavenging activities were found to be highly correlated with polyphenolic content. Thus, it was very important to determine if there was relevance between antioxidant capacity and phenolic content in this study. The radical scavenging capacity of MMEs might be related mostly to their phenolic hydroxyl groups. This correlation suggests that although MME may contain other antioxidants such as icariin, these contribute in a minor way, or its antioxidant activity is expressed in another way.

## 4. Conclusion

In our study, HPLC-DAD and HPTLC fingerprinting coupled with a chemical profiling method based on UHPLC-Q-Orbitrap-MS was applied to rapidly detect characteristic chemical markers for quality control and quantitative analysis of MMEs. At the same time, we tested the crude drug antioxidant activity using HPTLC-DPPH and HPTLC-ABTS^*∗*+^ experiments.

This newly constructed method has advantages in that the relatively routine and cheap HPTLC and HPLC-DAD fingerprinting approach could be used to analyse MMEs to quickly detect characteristic extraction peaks, whereas the UHPLC-Q-Orbitrap-MS method with a relatively high expense is only employed to quickly identify the characteristic peaks. Therefore, a quite cost effective tactic was developed for the rapid discovery of appropriate marker compounds for quality management of MMEs. For example, daidzein, gallic acid, and icariin were found to be the main characteristic components in MMEs. In addition, icariin could possibly be chosen as an appropriate qualitative and quantitative marker for evaluating the quality of MMEs. The results revealed that fingerprinting with integration of multiple targets revealed not only multiple suppressed activities but also chemical information regarding MMEs with multiple targets. Our experimental results regarding antioxidant activity clarified that HPTLC combined with ABTS^*∗*+^ and DPPH^•^ is a meaningful and powerful tool to comprehensively examine the inhibitory activity and potential antioxidants in conventional Uyghur medicines.

## Figures and Tables

**Table 1:** Raw material samples utilized in this work and their similarities.

Sample	Batch	Similarity
S1	20161213	0.998
S2	20170112	0.998
S3	20170122	0.999
S4	20170210	0.998
S5	20170220	0.981
S6	20170228	0.997
S7	20170315	0.998
S8	20170325	0.998
S9	20170328	0.998
S10	20170415	0.997

**Table 2:** Linearity of calibration curve.

Standard substance	Calibration curve	*R* ^2^	Linear range (*μ*g/ml)
Gallic acid	*Y* = 0.3011*X* + 0.0524	0.9999	5.568~33.408
Daidzein	*Y* = 0.3984*X* − 0.0005	1.0000	2.368~14.208
Icariin	*Y* = 0.2278*X* + 0.0173	1.0000	20.640~123.840

*Y* represents UV absorbency or peak area in DAD profiles, and *X* represents injected compound concentration.

**Table:** (a) The RRT and RPA of precision, repeatability, and stability of the three marker compounds

Name	Standard substance	Precision (*n* = 6)		Repeatability (*n* = 10)		Stability (*n* = 10)	
		Mean	RSD (%)	Mean	RSD (%)	Mean	RSD (%)
RSD of RRT (%)	Gallic acid	10.90	0.004	10.91	0.002	10.91	0.001
	Daidzein	34.71	0.005	34.82	0.001	34.84	0.000
	Icariin	36.09	0.002	36.12	0.003	36.10	0.000
RSD of RPA (%)	Gallic acid	4.18	0.027	3.96	0.057	4.00	0.005
	Daidzein	2.34	0.0021	2.31	0.053	2.39	0.003
	Icariin	11.76	0.0015	12.76	0.033	12.83	0.003

**Table:** (b) Recovery of the three markers in ten batches of MME

Standard substance	Recovery (%) (*n* = 10)	
	Mean	RSD (%)
Gallic acid	100.68	2.75
Daidzein	100.56	2.91
Icariin	100.49	1.89

**Table 4:** Contents (%) of the three bioactive chemical compounds in 10 batches of MMEs.

Sample number	Batch number	Gallic acid content (%)	Daidzein content (%)	Icariin content (%)
S1	20161213	0.1159	0.0407	0.6076
S2	20170112	0.1122	0.0397	0.5903
S3	20170122	0.1186	0.0417	0.6218
S4	20170210	0.1345	0.0490	0.6300
S5	20170220	0.1196	0.0485	0.6254
S6	20170228	0.1455	0.0498	0.6500
S7	20170315	0.1256	0.0542	0.6423
S8	20170325	0.1289	0.0588	0.6251
S9	20170328	0.1254	0.0591	0.6231
S10	20170415	0.1356	0.0542	0.6444

Average content (%)		0.1262	0.0496	0.6260

RSD (%)		0.0811	0.1440	0.0284

**Table 5:** Compounds identified in MME via UHPLC-DAD-Quadrupole-Orbitrap-MS.

Peak number	Identified	*t*_R_/min	Molecular formula	[M − H]^−^ [M + H]^+^			Fragment ion *m*/*z*
				Mean measured mass (Da)	Theoretical exact mass (Da)	Mass accuracy	
5	Gallic acid	9.23	C_7_H_6_O_5_	169.01308 [M − H]^−^	170.12	−0.40093	69.03307/97.02800/125.02302
8	Salidroside	15.96	C_14_H_20_O_7_	299.11285 [M − H]^−^	300.3044	1.06289	59.01240/71.01236/78.95756/89.02291/119.04881
11	Catechin	18.33	C_15_H_14_O_6_ (^∙^H_2_O)	289.07101 [M − H]^−^	290.27	1.19317	78.95754/96.95860/107.01235/125.02298/169.01219
12	Chlorogenic acid	18.90	C_16_H_18_O_9_	353.08684 [M − H]^−^	354.31	0.36991	85.02798/191.05502
17	Rutinum	28.35	C_27_H_30_O_16_	609.144431 [M − H]^−^	610.51	4.52846	271.02411/300.02667
18	Ferulic acid	29.28	C_10_H_10_O_4_	193.04959 [M − H]^−^	194.19	0.28054	78.95752/96.96812/139.02851
19	Hyperoside	29.35	C_21_H_20_O_12_	463.08725 [M − H]^−^	464.3763	0.31077	271.02423/300.02692
20	Luteoloside	33.05	C_21_H_20_O_11_	447.09217 [M − H]^−^	448.3769	−0.04172	271.02420/300.02679
22	Daidzein	40.28	C_15_H_10_O_4_	255.06542 [M + H]^+^	254.24	0.93679	91.05470/137.02321/181.06456/199.07507
25	Quercetin	43.18	C_15_H_10_O_7_	301.03458 [M − H]^−^	302	0.94466	78.95753/107.01234/121.02807/151.00235/178.99734/202.93114
26	Icariin	43.90	C_33_H_40_O_15_	677.24431 [M + H]^+^	676.6617	−0.77767	531.1873/515.1931/369.1336/313.07080
27	Apigenin	48.39	C_15_H_10_O_5_	269.04486 [M − H]^−^	270.23	1.52100	117.03310/151.00232/107.01233
29	Kaempferol	50.01	C_15_H_10_O_6_	285.03971 [M − H]^−^	286.23	1.20869	78.95751/93.03302/150.01020/190.015014

## References

[1] Standard for Preparations of Uyghur Medical Institutions in Xinjiang Uyghur Autonomous Region, Urumqi, China, 1999.

[2] Lone S. A., Kushwaha M., Wani A. (2017). Genetic diversity, LCMS based chemical fingerprinting and antioxidant activity of Epimedium elatum Morr & Decne. *Journal of Applied Research on Medicinal and Aromatic Plants*.

[3] Jiang J., Song J., Jia X. (2015). Phytochemistry and ethnopharmacology of epimedium L. species. *Chinese Herbal Medicines*.

[4] Ahmad A., Tandon S., Xuan T. D., Nooreen Z. (2017). A review on phytoconstituents and biological activities of cuscuta species. *Biomedicine & Pharmacotherapy*.

[5] Edouard M. J., Miao L., Fan G.-W. (2014). Yang-tonifying traditional Chinese medicinal plants and their potential phytoandrogenic activity. *Chinese Journal of Natural Medicines*.

[6] Panossian A., Wikman G., Sarris J. (2010). Rosenroot (*Rhodiola rosea*): traditional use, chemical composition, pharmacology and clinical efficacy. *Phytomedicine*.

[7] Pang W., Yang H., Wu Z., Huang M., Hu J. (2009). LC-MS-MS in MRM mode for detection and structural identification of synthetic hypoglycemic drugs added illegally to 'natural' anti-diabetic herbal products. *Chromatographia*.

[8] Peres R. G., Tonin F. G., Tavares M. F. M., Rodriguez-Amaya D. B. (2013). HPLC-DAD-ESI/MS identification and quantification of phenolic compounds in ilex paraguariensis beverages and on-line evaluation of individual antioxidant activity. *Molecules*.

[9] Fan X. H., Wang Y., Cheng Y. Y. (2006). LC/MS fingerprinting of Shen mai injection: a novel approach to quality control of herbal medicines. *Journal of Pharmaceutical & Biomedical Analysis*.

[10] Li S. P., Zhao J., Yang B. (2011). Strategies for quality control of Chinese medicines. *Journal of Pharmaceutical Biomedical Analysis*.

[11] Yi L.-Z., Yuan D.-L., Liang Y.-Z., Xie P.-S., Zhao Y. (2007). Quality control and discrimination of *Pericarpium Citri Reticulatae* and *Pericarpium Citri Reticulatae Viride* based on high-performance liquid chromatographic fingerprints and multivariate statistical analysis. *Analytica Chimica Acta*.

[12] Zheng G., Yang D., Wang D., Zhou F., Yang X., Jiang L. (2009). Simultaneous determination of five bioactive flavonoids in Pericarpium Citri Reticulatae from China by high-performance liquid chromatography with dual wavelength detection. *Journal of Agricultural and Food Chemistry*.

[13] Yang Y., Jiang L., Zheng G.-D., Lin L.-W., Chen J.-L., Zhou W. (2011). HPLC fingerprint of pericarpium citri reticultae from Guangdong province. *Journal of Chinese Medicinal Materials*.

[14] Zhang X.-L., Liu L.-F., Zhu L.-Y. (2014). A high performance liquid chromatography fingerprinting and ultra high performance liquid chromatography coupled with quadrupole time-of-flight mass spectrometry chemical profiling approach to rapidly find characteristic chemical markers for quality evaluation of dispensing granules, a case study on Chuanxiong Rhizoma. *Journal of Pharmaceutical and Biomedical Analysis*.

[15] Sukumar D., Arimboor R., Arumughan C. (2008). HPTLC fingerprinting and quantification of lignans as markers in sesame oil and its polyherbal formulations. *Journal of Pharmaceutical and Biomedical Analysis*.

[16] Zarzycki P. K., Ślaogonekczka M. M., Zarzycka M. B., Włodarczyk E., Baran M. J. (2011). Application of micro-thin-layer chromatography as a simple fractionation tool for fast screening of raw extracts derived from complex biological, pharmaceutical and environmental samples. *Analytica Chimica Acta*.

[17] Milojković-Opsenica D., Ristivojević P., Andrić F., Trifković J. (2013). Planar chromatographic systems in pattern recognition and fingerprint analysis. *Chromatographia*.

[18] Yang T., Zhang S., Wang R. (2016). Polysaccharides from rhizoma panacis majoris and its anti-oxidant activity. *International Journal of Biological Macromolecules*.

[19] Chen X., Guo Y., Hu Y., Yu B., Qi J. (2016). Quantitative analysis of highly similar salvianolic acids with 1H qNMR for quality control of traditional Chinese medicinal preparation Salvianolate Lyophilized Injection. *Journal of Pharmaceutical and Biomedical Analysis*.

[20] Kedare S. B., Singh R. P. (2011). Genesis and development of DPPH method of antioxidant assay. *Journal of Food Science and Technology*.

[21] Snezana A. K. D. W. (2015). Thin-layer chromatography-bioassay as powerful tool for rapid identification of bioactive components in botanical extracts. *Modern Chemistry & Applications*.

[22] Cieśla Ł., Kryszeń J., Stochmal A., Oleszek W., Waksmundzka-Hajnos M. (2012). Approach to develop a standardized TLC-DPPH test for assessing free radical scavenging properties of selected phenolic compounds. *Journal of Pharmaceutical and Biomedical Analysis*.

[23] Agatonovic-Kustrin S., Babazadeh Ortakand D., Morton D. W., Yusof A. P. (2015). Rapid evaluation and comparison of natural products and antioxidant activity in calendula, feverfew, and German chamomile extracts. *Journal of Chromatography A*.

[24] Takamatsu S., Hodges T. W., Rajbhandari I., Gerwick W. H., Hamann M. T., Nagle D. G. (2003). Marine natural products as novel antioxidant prototypes. *Journal of Natural Products*.

[25] Agatonovic-Kustrin S., Morton D. W., Yusof A. P. (2016). Development and validation of a simple high performance thin layer chromatography method combined with direct 1,1-diphenyl-2-picrylhydrazyl assay to quantify free radical scavenging activity in wine. *Food Chemistry*.

[26] Agatonovic-Kustrin S., Hettiarachchi C. G., Morton D. W., Razic S. (2015). Analysis of phenolics in wine by high performance thin-layer chromatography with gradient elution and high resolution plate imaging. *Journal of Pharmaceutical and Biomedical Analysis*.

[27] Bandonienappproaches the limit D., Murkovic M. (2002). The detection of radical scavenging compounds in crude extract of borage (Borago officinalis L.) by using an on-line HPLC-DPPH method. *Journal of Biochemical and Biophysical Methods*.

[28] Olech M., Komsta Ł., Nowak R., Cieśla Ł., Waksmundzka-Hajnos M. (2012). Investigation of antiradical activity of plant material by thin-layer chromatography with image processing. *Food Chemistry*.

[29] Santos D. N. E., Souza L. L. D., Ferreira N. J., Oliveira A. L. D. (2015). Study of supercritical extraction from brazilian cherry seeds (Eugenia uniflora L.) with bioactive compounds. *Food and Bioproducts Processing*.

[30] Rossi D., Guerrini A., Maietti S. (2011). Chemical fingerprinting and bioactivity of Amazonian Ecuador *Croton lechleri* Müll. Arg. (Euphorbiaceae) stem bark essential oil: a new functional food ingredient?. *Food Chemistry*.

[31] Chen J., Song Y., Li P. (2007). Capillary high-performance liquid chromatography with mass spectrometry for simultaneous determination of major flavonoids, iridoid glucosides and saponins in Flos Lonicerae. *Journal of Chromatography A*.

[32] Jin Y., Liang T., Fu Q. (2009). Fingerprint analysis of Ligusticum chuanxiong using hydrophilic interaction chromatography and reversed-phase liquid chromatography. *Journal of Chromatography A*.

[33] Li Y., Wu T., Zhu J. (2010). Combinative method using HPLC fingerprint and quantitative analyses for quality consistency evaluation of an herbal medicinal preparation produced by different manufacturers. *Journal of Pharmaceutical & Biomedical Analysis*.

[34] Jiang Y., Li S. P., Wang Y. T., Chen X. J., Tu P. F. (2009). Differentiation of Herba Cistanches by fingerprint with high-performance liquid chromatography-diode array detection-mass spectrometry. *Journal of Chromatography A*.

[35] Szewczyk K., Olech M. (2017). Optimization of extraction method for LC–MS based determination of phenolic acid profiles in different Impatiens species. *Phytochemistry Letters*.

[36] Abdulla R., Mansur S., Lai H. (2017). Qualitative analysis of polyphenols in macroporous resin pretreated pomegranate husk extract by HPLC-QTOF-MS. *Phytochemical Analysis*.

[37] Zhu J., Yi X., Zhang J., Chen S., Wu Y. (2017). Chemical profiling and antioxidant evaluation of Yangxinshi Tablet by HPLC–ESI-Q-TOF-MS/MS combined with DPPH assay. *Journal of Chromatography B*.

[38] Ren Q., Long S. (2017). Chemical identification and quantification of Hu-Gu capsule by UHPLC–Q-TOF-MS and HPLC-DAD. *Revista Brasileira de Farmacognosia*.

[39] Ristivojević P., Trifković J., Vovk I., Milojković-Opsenica D. (2017). Comparative study of different approaches for multivariate image analysis in HPTLC fingerprinting of natural products such as plant resin. *Talanta*.

[40] Cheng S.-C., Huang M.-Z., Shiea J. (2011). Thin layer chromatography/mass spectrometry. *Journal of Chromatography A*.

